# The positive correlation of TIPRL with LC3 and CD133 contributes to cancer aggressiveness: potential biomarkers for early liver cancer

**DOI:** 10.1038/s41598-019-53191-5

**Published:** 2019-11-14

**Authors:** Soo Young Jun, Su-Jin Jeon, Ji-Yong Yoon, Jeong-Ju Lee, Hyang Ran Yoon, Min-Hyuk Choi, Debasish Halder, Kwangho Lee, Nam-Soon Kim

**Affiliations:** 1 Medical Genomics Research Center, Korea Research Institute of Bioscience & Biotechnology, Daejeon, 34141 Republic of Korea; 2 Rare-Disease Research Center, Division of Strategic Research Groups, Korea Research Institute of Bioscience & Biotechnology, Daejeon, 34141 Republic of Korea; 3 Immunotherapy Convergence Research Center, Korea Research Institute of Bioscience & Biotechnology, Daejeon, 34141 Republic of Korea; 4 Bio and Drug Discovery Division, Korea Research Institute of Chemical Technology, Daejeon, 34114 Republic of Korea; 5 Functional Genomics, University of Science & Technology, Daejeon, 34113 Republic of Korea

**Keywords:** Liver cancer, Tumour biomarkers

## Abstract

Studies have reported dysregulation of TIPRL, LC3 and CD133 in liver cancer tissues. However, their respective relationships to liver cancer and roles as biomarkers for prognosis and diagnosis of liver cancer have never been studied. Here we report that the level of TIPRL is significantly correlated with levels of LC3 (Spearman *r* = 0.9) and CD133 (*r* = 0.7) in liver cancer tissues. We observed significant upregulations of TIPRL, LC3 and CD133 in hepatocellular carcinomas (HCCs) compared with adjacent normal tissues. Importantly, TIPRL, tested among additional variables, showed a significant impact on the prognosis of HCC patients. TIPRL knockdown significantly reduced expressions of *LC3*, *CD133*, stemness-related genes, as well as viability and stemness of liver cancer cell-lines, which were promoted by ectopic *TIPRL* expression. Either alone or as a combination, TIPRL, LC3 and CD133 showed significant values of area under the curve (AUC) and sensitivity/specificity in early liver cancer tissues. Furthermore, the statistical association and the diagnostic efficacies of TIPRL, LC3 and CD133 in HCC tissues were confirmed in a different IHC cohort. This data demonstrates that the complex involvement of TIPRL/LC3/CD133 in liver cancer aggressiveness can together or individually serve as potential biomarkers for the early detection of liver cancer.

## Introduction

As the second leading cause of death worldwide, cancer causes about 1 in 6 deaths. There were an estimated 782,000 reports of liver cancer related deaths in 2018, according to the World Health Organization (WHO)^1^. Despite rapid advances in liver cancer diagnosis and therapy in recent decades, liver cancer death rates have increased 3% per year since 2000. Public health agencies, such as WHO, have reported that this increase is mainly caused by late-stage presentation and inaccessible diagnosis and treatment: contrary to 90% of high-income countries, only 26% of low-income countries have general pathology services in the public sector^2^. Thus, there is an urgent demand to develop diagnostic resources for monitoring in high risk populations, which could provide information to clinicians for potential curative intervention, many of which require early tumor identification.

The human TOR signaling regulator (hereafter TIPRL) protein is the mammalian ortholog of the yeast TIP41 protein, which was originally reported as a binding partner for type 2A-associated protein of 42 kilodaltons (Tap42). TIPRL interacts with protein phosphatase type2Ac (PP2Ac), thereby inhibiting its activity to oppose ataxia telangiectasia mutated (ATM) and ATM and Rad3-related (ATR) dependent phosphorylation events; while not interacting with α4, which is the mammalian homolog of Tap42, unlike the yeast TIP41^3^. Previously, our team reported the TIPRL protein contributing to TNF-related apoptosis inducing ligand (TRAIL) resistance of liver cancers^4^. TIPRL is highly upregulated in hepatocellular carcinomas (HCCs) compared with the adjacent liver tissues, and negatively regulates the MKK7/JNK axis, thereby preventing prolonged activation of MKK7 and JNK and, as a consequence, TRAIL-induced apoptosis.

CD133 (also called Prominin-1) has been used for identifying tumor initiating cells or cancer stem cells (CSCs)^5^. CD133 + HCCs have been reported to exhibit stem-like properties, such as a xenograft showing histological resemblance with the parent tumor, self-renewal ability, and the generation of daughter cells possessing some proliferative capacity. Antibodies targeting CD133 have shown an inhibition of cell proliferation^6^. γ-irradiation caused resistance of CD133 + glioma cells by induction of autophagy that can be inhibited by the autophagy inhibitor^7^. Furthermore, Chen *et al*.^8^, demonstrated that CD133 actively relocated from the plasma membrane into the cytoplasm, and this re-location caused autophagy in HCCs. Consistently, CD133 showed a strong association with microtubule-associated protein light chain 3 (LC3), an autophagy marker, during glucose starvation, collectively indicating the involvement of CD133 in survival and stemness properties of HCC cells.

Liver malignancy is exacerbated by production of hepatic cancer stem cells through autophagy induction^9^. Autophagy, more specifically mitophagy, contributes to production and maintenance of hepatic cancer stem cells as well as promotion of hepatocarcinogenesis by suppression of TP53 and induction of the expression of the transcription factor NANOG. Thus, although its role as tumor suppressor^10^ in nontumor cells or the early stage of tumor cell development has been reported, autophagy also plays a key role in cancer cell survival once tumors, including HCCs, are established. Nevertheless, fundamental roles of autophagy in aggressiveness of liver cancers are still obscure. In this study, we attempted to study relationships between TIPRL, LC3 and CD133, which individually has been reported in autophagy and liver aggressiveness, using human liver tissues, including HCCs, and HCC/liver cancer cell-lines. Here we report the interrelationships between TIPRL, LC3 and CD133 in HCC/liver cancer tissues are associated with cancer aggressiveness via possibly induction of cancer cell stemness, thereby providing novel biomarkers for early prognosis and diagnosis of liver cancer.

## Results

### TIPRL is upregulated in HCCs and predicts a poor prognosis of HCC patients

**Figure 1:**
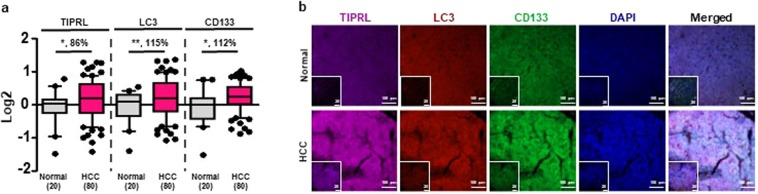
Upregulations of TIPRL, LC3 and CD133 in HCCs. Human tissues were stained with the indicated antibodies followed by confocal observation. **(a)** The levels of TIPRL, LC3 and CD133 were determined using the ZEN program (Supplementary Tables 1 and 2). *p*-values (^*^P < 0.05; ^**^P < 0.01) were determined by a paired t-test, and % differences are shown. (n) is the number of samples. **(b)** The representative images were selected from normal and HCCs groups, respectively. DAPI was used for nucleus staining, and scar bars, 20 (inserted) and 100 μm.

Given our previous report that TIPRL confers a TRAIL resistant property to hepatocellular carcinomas (HCCs)^4^, we examined levels of TIPRL as well as LC3^11^ and CD133^12^, which are markers for autophagy and cancer stem cells (CSCs), respectively: all contributing to chemo- and radio-resistance in liver cancers. For these, we stained human liver tissues, including HCCs (Supplementary Table 1) with the indicated antibodies and the levels of TIPRL, LC3 and CD133 were determined following normalization of raw data obtained using confocal observation and the ZEN program (Methods; Supplementary Table 2). Consistent with our previous report^4^, we observed significant upregulation of TIPRL in HCCs compared with the adjacent liver tissues (Fig. 1a,b and Supplementary Fig. 1a–d). LC3 and CD133 were strikingly upregulated in HCCs in contrast to normal tissues. We confirmed the upregulations of *TIPRL* and *LC3* mRNA in public datasets (www.oncomine.org, Supplementary Figs. 1e,f).

**Figure 2:**
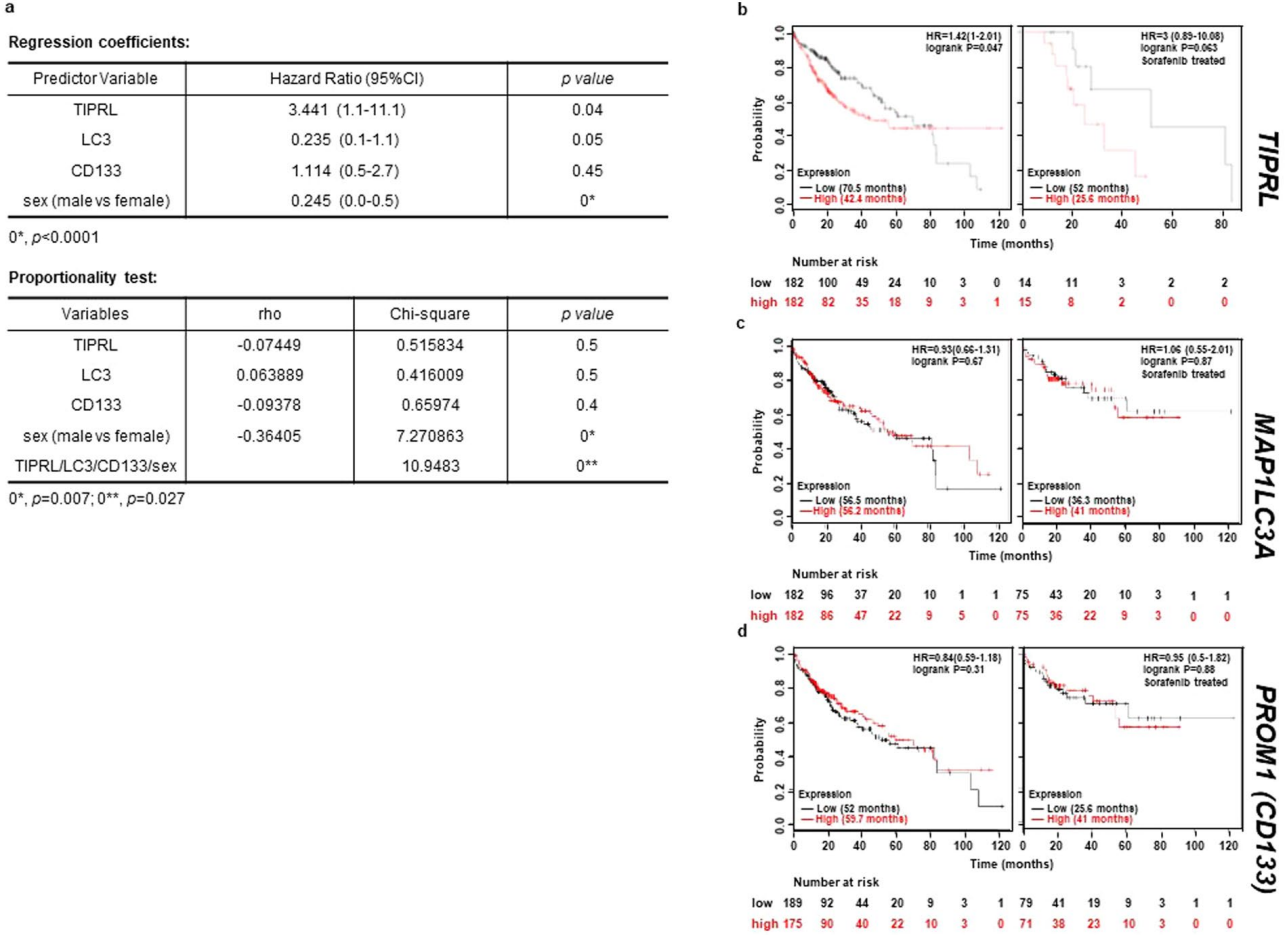
TIPRL is associated with the poor prognosis of HCC patients. (**a**) A Cox regression hazard coefficients (upper) and a proportionality test (lower) were performed to determine the hazard/prognostic effect and independency of the variables, TIPRL, LC3, CD133, sex (male vs female), on the survivability of HCC patients using XLSTAT. **(b**–**d)** A public database (www.kmplot.com) was used to examine relationships between mRNA levels of *TIPRL*, *MAP1LC3A* (LC3) and *PROM1* (CD133) and survivability of HCC patients.

Next, we merged the variables including TIPRL, LC3, CD133 level and the clinical parameter sex into a multivariate Cox proportional hazards regression model to correct for confounding variables and to determine the independent effects of risk factors (Fig. 2a, upper). Among the four variables, TIPRL (HR 3.4 95%CI = 1.1–11.1, *p* = 0.04) exhibits significant prognostic influence on HCC patients but LC3 (HR 0.2 95%CI = 0.1–1.1, *p* = 0.05), CD133 (HR 1.1 95%CI = 0.5–2.7, *p* = 0.45) and sex (HR 0.2 95%CI = 0.0–0.5, *p* < 0.0001) do not. Furthermore, the covariates TIPRL and LC3, among additional variables, show that their *p*-values are greater than α-0.05 in the proportionality test, indicating that TIPRL and LC3 can solely explain an HCC incidence (Fig. 2a lower).

To further support this analysis, we used the public database (www.kmplot.com)^13^ and studied the relationships between levels of *TIPRL*, *LC3* and *CD133* and the overall survival (OS) of HCC patients. In keeping with the multivariate Cox model, *TIPRL* significantly influenced the OS of patients in both a whole population of HCC (HR 1.42, logrank *p* = 0.05) and sorafenib-treated patients (HR 3, logrank *p* = 0.06), which is currently the only systemic agent approved for use in HCCs; however, *MAP1LC3A* (HR 0.93, logrank *p* = 0.7; HR = 1.1, logrank *p* = 0.9) and *PROM1* (*CD133*; HR 0.8, logrank *p* = 0.3; HR 1, logrank *p* = 0.9) did not show significant influence (Fig. 2b–d). Moreover, *TIPRL* exhibited a more enhanced HR ratio in the sorafenib-treated group than a whole population group of HCCs suggesting that TIPRL as an independent risk factor has significant prognostic influence on HCCs related to drug resistance.

### TIPRL is required for liver cancer cell survival and stemness

**Figure 3:**
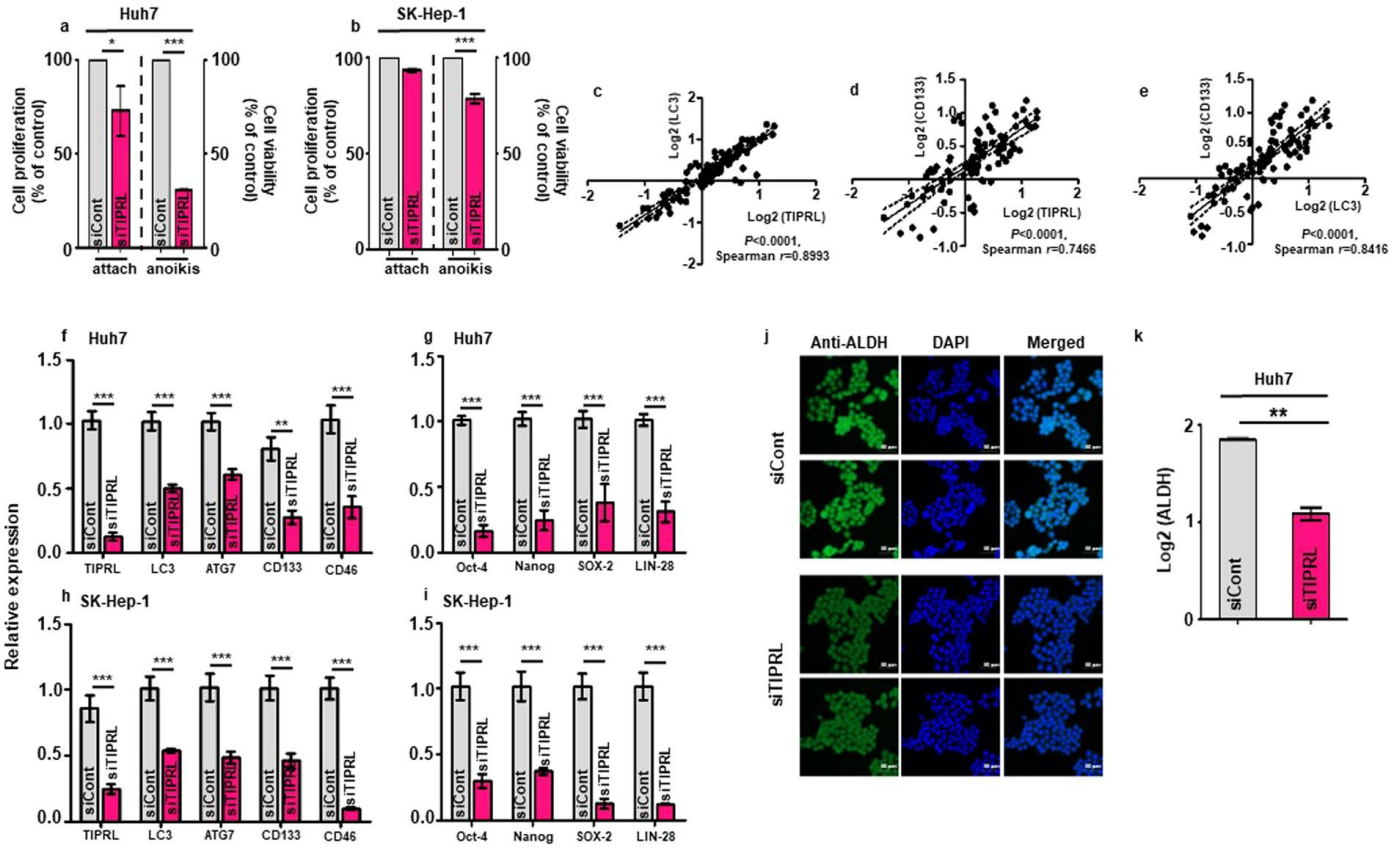
TIPRL is an essential element for liver cancer cell survival and stemness. Huh7 (**a**,**f**,**g,j,k)** and SK-Hep-1 **(b**,**h**,**i)** cells were transfected with siCont/siTIPRL. After 72 hours, MTT analyses were performed to determine both the proliferation index (**a**,**b** left) and survival ratio **(a**,**b** right) of the cells. **(c**–**e)** Spearman correlation was used to determine correlations between levels of TIPRL, LC3 and CD133 in HCC tissues. Each dot represents a single sample. **(f**–**i)** 72 hours after siTIPRL transfection, quantitative RT-PCR analyses were performed to determine the mRNA levels of the indicated genes using primers (Supplementary Table 3). **(j)** Immunocytochemistry was performed using an anti-ALDH antibody to determine the level of ALDH activity in siCont/siTIPRL-transfected Huh7 cells. For nucleus staining, DAPI was used, and scar bar, 50 μm. **(k)** Quantification of ALDH activity observed in **(j)**. All experiments were independently repeated four times. Statistical differences (^*^P < 0.05; **P < 0.01; ***P < 0.0001) were determined by 2way ANOVA **(a**,**b)**, paired t-test (**f**–**i**), and an unpaired t-test (**k**).

Next, we studied the roles of TIPRL in an HCC incidence. MTT assays show that TIPRL knockdown reduced cell proliferation in an attached condition and, significantly, the viability of Huh7 and SK-Hep-1 cells in an anoikis (Fig. 3a,b). TIPRL was originally determined as a negative regulator of the catalytic subunits of Type 2A phosphatases (PP2Ac)^3^, and the complex relationship between TIPRL, PP2Ac and mTOR has been recently reported^14^. Considering that mTOR is a master regulator of autophagy contributing to cancer cells’ survival via promoting stemness^15^, we examined possible associations of TIPRL, LC3 and CD133 levels in HCC tissues. We found statistically significant associations of TIPRL with LC3 and CD133; when the level of TIPRL was increased, both expressions of LC3 and CD133 were correspondingly augmented, as shown by significant values of Spearman *r* (Fig. 3c–e). In addition, the LC3 level was statistically correlated with the CD133 level.

Given this significant correlation between levels of TIPRL, LC3 and CD133 in HCC tissues, we investigated and observed significant reductions in *TIPRL*, *LC3*, *ATG7*, *CD133* and *CD46* mRNA levels in HCC/liver cancer cell-lines transfected with two different small interfering RNA against TIPRL (siTIPRL) (Fig. 3f,h and Supplementary Fig. 2a,c). In agreement with their respective relationships to liver cancer, TIPRL knockdown strikingly decreased mRNA expressions of *Oct-4*, *Nanog*, *SOX-2* and *LIN-28*, stemness related genes^16^ (Fig. 3g,i and Supplementary Fig. 2b,d). This was further supported by the observations that TIPRL knockdown decreased LC3 and CD133 expression while CD133 knockdown failed to reduce TIPRL and LC3. Moreover, ectopic *TIPRL* promoted expressions of LC3 and CD133 as well as viability of HCC/liver cancer cell-lines, which were reduced in siTIPRL/siCD133-cells (Supplementary Fig. 2e–j). Furthermore, these reductions were in keeping with significantly reduced activity of aldehyde dehydrogenase (ALDH) used to discriminate the CD133 liver cancer stem cell population, in siTIPRL-Huh7 cells, compared with the activity in siCont-cells (Fig. 3j,k). Overall, this data indicates that, as an upstream modulator of LC3 and CD133, which are involved in tumor aggressiveness, TIPRL is a critical player in HCC/liver cancer cell proliferation, viability and stemness, which are key events for HCC incidence and progression.

### Significant association of TIRPL, LC3 and CD133 levels in liver cancer tissues

**Figure 4:**
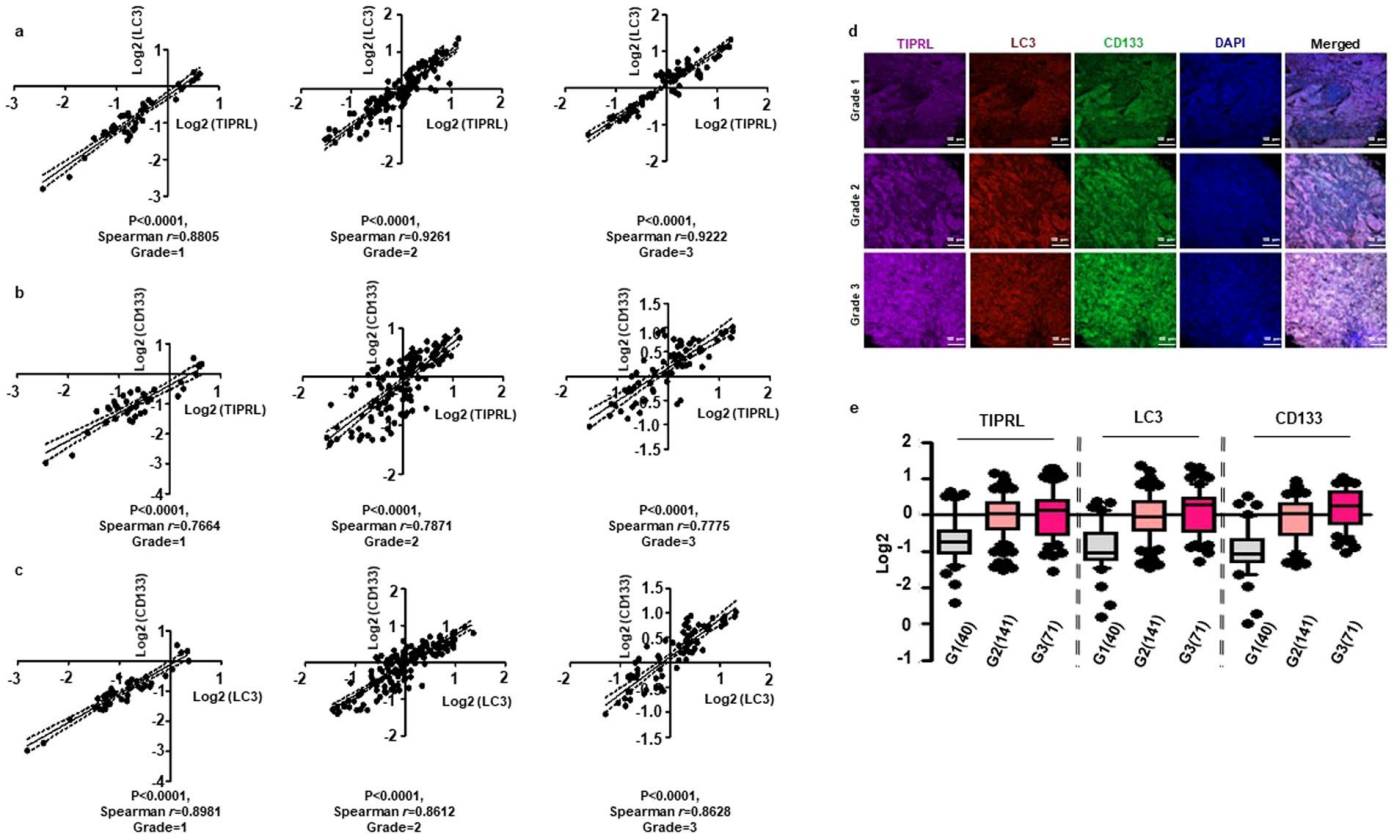
Significant correlations between levels of TIPRL, LC3 and CD133 in liver cancers. (**a**–**c)** The statistically significant associations between levels of TIPRL, LC3 and CD133 in each grade of liver cancers were determined by the Spearman correlation. Each dot indicates a single patient. **(d)** The representative images were selected from each grade of human liver cancer tissues that were stained with indicated antibodies. DAPI was used for nucleus staining, and scar bar, 100 μm. **(e)** Quantitative analyses of TIPRL, LC3 and CD133 were conducted according to each grade of liver cancers. (n), the number of samples is indicated.

To extend our observation that the expression of TIPRL is associated with levels of LC3 and CD133 in HCC tissues (Fig. 3c–e), we analyzed the statistical relationship between their levels in the following liver cancer tissues according to cancer grade: hepatocellular carcinoma, intrahepatic cholangiocarcinoma, adenosquamous carcinoma, carcinoid and mixed carcinoma, comprised of intrahepatic cholangiocarcinoma and hepatocellular carcinoma (Supplementary Table 2). We determined significant correlations between levels of TIPRL, LC3 and CD133, as calculated by significant values of Spearman *r*, in each grade of liver cancers. Moreover, we observed that each correlation line moves from left to right in the progression of liver cancers (Fig. 4a–c). Confocal observation (Fig. 4d), as well as statistical analysis (Fig. 4e) consistently confirm gradual increases of TIPRL, LC3 and CD133 levels according to liver cancer progression, suggesting critical roles of TIPRL, LC3 and CD133 association in the progression of liver cancers.

### Diagnostic power of TIPRL, LC3 and CD133 expression

**Figure 5:**
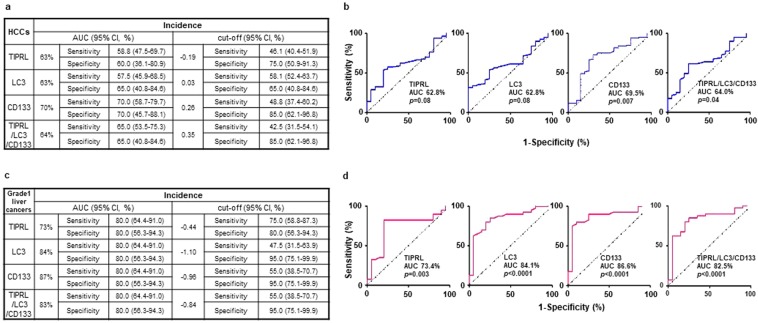
Diagnostic efficacies of TIPRL, LC3, CD133 and the TIPRL/LC3/CD133 models in HCCs and early liver cancers. Diagnostic efficacies of TIPRL, LC3, CD133 and the combined models in HCCs (**a**,**b**) and grade1 liver cancers **(c**,**d)** were determined using ROC analysis. AUC, area under the curve.

We attempted to analyze diagnostic potentials of TIPRL, LC3, CD133 and the TIPRL/LC3/CD133 levels in liver cancers. The ROC analyses exhibited approximately 50% of are under the curve (AUC) in four models; in addition, the *p* values were not significant (Supplementary Fig. 3), and when calculated based on the Youden and likelihood index, the cut-off of four models had low sensitivity. The TIPRL/LC3/CD133 model did not increase the diagnostic power of TIPRL, LC3 and CD133 (Supplementary Fig. 3). We then calculated diagnostic potentials of TIPRL, LC3, CD133 and the combined level in HCCs (Fig. 5a,b). Statistical analysis of four models in HCC tissues show significant increases in AUC ratios and corresponding *p*-values, respectively, compared to the ratios and *p*-vales in a whole collection of liver cancers (TIPRL and LC3, AUC 62.8%, *p* = 0.08; CD133, AUC 69.5%, *p* = 0.007; TIPRL/LC3/CD133, AUC 64.%, *p* = 0.04). Moreover, the cut-off of each model shows increased sensitivity with reasonable ranges of 95% CI (TIPRL, Sensitivity 46.1%, 95%CI = 40.4–51.9; LC3, Sensitivity 58.1, 95%CI = 52.4–63.7; CD133, Sensitivity 48.8, 95%CI = 37.4–60.2; TIPRL/LC3/CD133, Sensitivity 42.5, 95%CI = 31.5–54.1), thus implying improved diagnostic efficiencies of the TIPRL, LC3, CD133 and TIPRL/LC3/CD133 models in HCCs rather than in a whole population of liver cancers.

Considering the limited sensitivity for early detection of HCCs/liver cancers of current screening methodologies, such as computed tomography and biopsy, developing early biomarkers that could predict disease outcome is urgently needed. Thus, we analyzed early diagnostic potentials of TIPRL, LC3, CD133 and the TIPRL/LC3/CD133 models in grade 1 tissues of liver cancers. All groups show statistically significant AUCs and corresponding *p-*values (TIPRL, AUC 73%, *p* = 0.003; LC3, AUC 84%, *p* < 0.0001; CD133, AUC 87%, *p* < 0.0001; TIPRL/LC3/CD133 AUC 83%, *p* < 0.0001). Furthermore, all exhibits statistically significant sensitivities and specificities as well as cut-off values with reasonable ranges of 95% CI (Fig. 5c,d). Together, this suggests the critical diagnostic potential of TIPRL, LC3, CD133 and TIPRL/LC3/CD133 models as biomarkers for early liver cancers.

### Reproducibility and reliability of TIPRL, LC3 and CD133 as HCC biomarkers

To further confirm whether the statistical correlation and prognostic/diagnostic efficacies of levels of TIPRL, LC3 and CD133 are reproducible, a different set of human tissues was stained with the indicated antibodies, and then the expression of each protein was determined (Supplementary Tables 4 and 5). In addition, CD46, known to be implicated in liver carcinogenesis^17^, was tested for its level and prognostic/diagnostic efficacies in HCC tissues as a control model.

**Figure 6:**
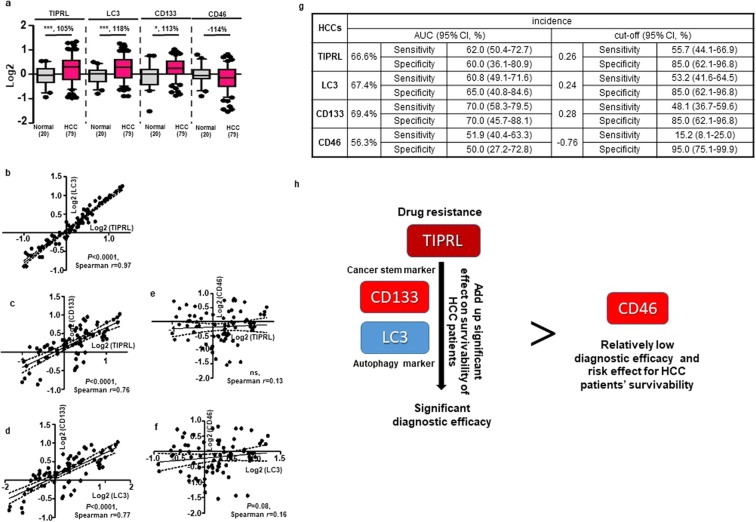
Confirmation of reproducibility of TIPRL, LC3 and CD133 as HCC biomarkers. (**a**) Using a different IHC cohort (Supplementary Tables 4 and 5), the levels of TIPRL, LC3, CD46 and CD133 were quantified using the ZEN program. Statistical differences (*P < 0.05; ***P < 0.0001) were calculated by a paired t-test, and % differences are shown. (n), the number of samples is indicated. **(b**–**f)** The correlations between levels of TIPRL, LC3, CD46 and CD133 were determined by the Spearman correlation equation. Single dots indicate an individual sample. **(g)** Diagnostic efficacies of TIPRL, LC3, CD133 and CD46 were determined by the ROC analysis. AUC, area under the curve. **(h)** Our study shows that the combination of TIPRL contributing to drug resistance with the cancer stem marker CD133 and autophagy marker LC3 produces novel HCC biomarkers. These biomarkers show more significant diagnostic efficacies than the CD46 currently being used as a liver cancer marker.

Consistent with the data (Fig. 1a,b), TIPRL, LC3 and CD133 were significantly upregulated in HCCs compared with the adjacent normal tissues, while CD46 was downregulated (Fig. 6a). Next, we confirmed the significant correlation of TIPRL and LC3, of TIPRL and CD133, as well as of LC3 and CD133 levels in HCC tissues, as exemplified by both significant Spearman *r* and corresponding *p* values (Fig. 6b–d). On the other hand, the correlations of TIPRL and CD46 as well as of LC3 and CD46 were not significant (Fig. 6e,f). Furthermore, TIPRL, LC3 and CD133 showed consistently higher values of AUC and sensitivity with reasonable ranges of 95% CI compared to CD46’s (Fig. 6g). This indicates more significant diagnostic efficacies of TIPRL, LC3 and CD133 than the efficacy of CD46, previously reported in liver carcinogenesis, for an HCC incidence (Fig. 6h).

Next, we performed a multivariate Cox proportional hazard analysis for TIPRL, LC3, CD46, CD133 and sex (male vs female) to determine the independent effects of risk factors in HCC patients (Supplementary Fig. 4). The regression coefficients show that all covariates except LC3 and sex, failed to gain significant *p*-values in the Cox analysis. However, LC3 did not present reasonable ranges of 95% CI. On the other hand, the sex and the TIPRL/LC3/CD46/CD133/sex models gained lower *p*-values than α-0.05 in a proportionality test, suggesting that these factors failed to independently explain survivability of HCC patients. Although it presented 0.058 as a *p*-value in the Cox regression analysis, TIPRL presents the most significant hazard ratio (HR 14.2). It also has the smallest values of rho and Chi-square (0.0061795 and 0.9961495, respectively), with 0.94 as a *p*-value in the proportionality test, together suggesting that TIRPL has a significant influential effect on the survivability of HCC patients, possibly as the sole risk factor.

## Discussion

The WHO predicts that there will be 1,341,344 liver cancer-related deaths in 2034 worldwide. Studies have reported that patients who were diagnosed early and received immediate treatment show higher survival than patients who were not^18^. However, liver cancers show no significant symptoms in early stages, and the patients usually visit physicians either at late stages of cancer or when comorbidities, such as liver cirrhosis, occur. Therefore, the discovery of biomarkers^19^ that can be used alone or with ultrasound scanning for the determination of early liver cancers are urgently required. Moreover, alpha-fetoprotein (AFP), which is a representable liver cancer biomarker, has reported poor diagnostic performance^20^; thus, the reliability of current liver cancer biomarkers are being questioned in clinical settings. Discovering novel biomarkers for liver cancer is a necessity; especially ones that are not influenced by other clinical parameters with both through determination of their roles as well as prognostic and diagnostic efficacies.

As an evolutionarily conserved pathway, autophagy is responsible for the recycling of bio-energetic components, such as damaged- or aged-cellular organelles and/or macromolecules. Given controversial reports^21,22^ in liver cancers, it is now accepted that autophagy has a dual role in liver cancer progression^23^. As an inhibitory role in the initial stages of liver tumorigenesis, autophagy decreases inflammation and/or preserves genome stability via limiting p62 accumulation^24^. On the other hand, in the later stages of liver tumorigenesis, autophagy exerts a pro-survival role to protect liver cancer cells against cell death caused by hypoxia^25^. This study determines the upregulation of TIPRL in HCCs, consistent with our previous report^4^, as well as an increase of LC3 and CD133 in tissues of HCCs, compared with the adjacent normal tissues. Furthermore, our study demonstrates the prominent prognostic effect of TIPRL on the HCC incidence and the patients’ survivability among other variables, including LC3, CD133, even CD46, and sex, which have reported their contributions in the HCCs incidence. This is supported by the observations that TIPRL knockdown inhibited survival and stemness efficacies of HCC/liver cancer cells via the reduction of autophagy, as well as the strong associations between levels of TIPRL, LC3 and CD133 in HCC and liver cancer tissues. Conversely, CD133 knockdown failed to reduce *TIPRL* and *LC3*. Furthermore, ectopic *TIPRL* promoted the LC3 and CD133 expression and viability of HCC/liver cancer cells. Overall, these results indicate TIPRL as a key modulator of LC3 and CD133 expressions in progress of liver cancer aggressiveness, and suggest novel links between autophagy and hepatocarcinogenesis.

Kimhofer *et al*.^26^, summarized several key selection criteria for an ideal biomarker as the following: targets should be measurable, have excellent prognostic and/or diagnostic abilities for conditions of interest (i.e., high sensitivity and specificity) and be amenable to measurement techniques that are reliable, reproducible and cheap, which provides accessibility to all populations. In addition, the techniques ideally take a simple kit-based format. Furthermore, biomarker candidates should be validated across a broad range of populations. Considering these criteria, we determined the strong correlation of TIPRL, LC3 and CD133 in a broad range of liver cancer tissues including HCCs. In addition, we observed statistically concurrent argumentation of TIPRL, LC3 and CD133 according to the progression of liver cancers, together indicating the complex interrelationships between levels of the biomarker candidates, TIPRL, LC3 and CD133 in liver cancer aggressiveness. Additionally, when their diagnostic potentials were analyzed in different subtypes of liver cancers, these candidates showed excellent diagnostic efficacies in tissues of grade1 liver cancers with significant sensitivity/specificity. Furthermore, we were able to determine the reliability and reproducibility of the early diagnostic efficacies of TIPRL, LC3 and CD133 candidates, compared to CD46 showing low ratios of AUC as well as sensitivity/specificity, in the different IHC cohort. Together, our study provides TIPRL, LC3 and CD133 as biomarkers for early liver cancers through determination of their roles as well as diagnostic efficacies in liver cancers.

The statistically significant association between the quantified results of the immunohistochemistry and the protein level of the targets has been reported in several studies using various methods, such as Western blotting and immune-enzymatic assays^27^. Compared to semi-quantitative methods based on visual scores, the computer-assisted image analyses^28^ have been reported to be superior in their quantification accuracy in multiple types of biomarkers. They thus complement the reproducibility and applicability of the semi-quantitative analysis, because they produce themselves to the desired quantitative results. In this study, human tissues were stained with the indicated antibodies followed by confocal observation, and then the levels of TIPRL, LC3, CD133 and CD46 were quantified using the ZEN program, provided by Carl Zeiss. Furthermore, we confirmed the reproducibility of the excellent diagnostic efficacies of TIPRL, LC3 and CD133 levels in HCCs at the different IHC cohort, thus providing reliability of TIPRL, LC3 and CD133 as novel biomarkers for HCCs and early liver cancers.

In conclusion, our study shows the statistical interrelationships between levels of TIPRL, LC3 and CD133 to HCC/liver cancer aggressiveness, and that prominent diagnostic efficacies of these proteins, either alone or a combination of TIPRL, LC3 and CD133, can be used as biomarkers for early diagnosis of liver cancer. Importantly, a Cox regression hazard model determined that TIPRL has a significant prognostic effect on the survivability of HCC patients, tested among additional variables, LC3, CD133 and sex. In keeping with this finding, TIPRL knockdown reduced survival and stemness efficacies of HCC/liver cancer cells via decreases in expressions of autophagy related- and of stemness related-genes. Ectopic *TIPRL* promoted the LC3 and CD133 expressions and viability of HCC/liver cancer cells. Furthermore, these significant associations and diagnostic efficacies of TIPRL, LC3 and CD133, especially in HCCs, were confirmed in the different IHC cohort. This study was based on the retrospective cohort study and a low number of samples. Thus, to be adapted to routine analysis to be used by clinicians to address prognostic of patients, our findings need to be further validated by prospective studies and clinical trials.

## Methods

### Patient Study: immunohistochemistry and histopathology

Patient samples (LV1221) were purchased from US Biomax (Rockville) and analyzed for the expression of TIPRL, LC3, CD133 and CD46 as the following; briefly, de-paraffinization was performed by immersing each slide in the order of xylene, as well as 100, 95, and 70% ethanol (Merck, 1.00983.1011) for five minutes each. Then the slides were subjected to a pre-heated sodium citrate buffer (0.01 M, pH 6.0; Merck, S4641) for 15 minutes to retrieve antigens. After that, the slides were incubated with antibodies against TIPRL (1:100; Bethyl laboratories, A300-663A), LC3 (1:100; Merck, L7543), CD133 (1:100; NOVUS, NBP2-37741) and CD46 (1:200; SCBT, sc-52647) overnight. The slides were then reacted with Alexa Fluor 633 goat anti-rabbit (1:100, TIPRL; Thermo Fisher Scientific, A11079), Alexa Fluor 568 goat anti-rabbit (1:100, LC3; Thermo Fisher Scientific, A21071) and Alexa Fluor 488 goat anti-mouse (1:100, CD133 and CD46; Thermo Fisher Scientific, T7458), respectively. Confocal observation (ZEISS LSM 800) was followed by quantification of each expression (Supplementary Table 2) using the Carl Zeiss LSM Image program (ZEN 2.3 lite) according to a previous study^29^.

Patient information provided by US Biomax (Rockville) was used for this study (Supplementary Tables 1 and 3).

### Cell culture and small-interfering RNAs (siRNAs) transfection

Human HCC, Huh7 (ThermoFisher Scientific) and liver adenocarcinoma cell line, SK-Hep-1 (ATCC) cells were authenticated by the Korean Collection for Type Cultures and maintained in DMEM and RPMI medium supplemented with 10% fetal bovine serum (FBS; Corning, 35-015-CV). Cells were regularly examined for *Mycoplasma* contamination.

For TIPRL knockdown, four different small-interfering RNAs based on TIPRL sequence (TIPRL, TIP41, NM_152902.3) were generated, and all showed over 90% TIPRL knockdown efficiencies. Among them, the #3 construct was mainly used for this study; 5′-CCUAAUGAAAUAUCCCAGUAUUU-3′ (sense), 5′-AUACUGGGAUAUUUCAUUAGGUU-3′ (anti-sense). The #2 was also used; 5′-CUGCCUCAGCCAUGAUGAU-3′ (sense), 5′-AUCAUCAUGGCUGAGGCAG-3′ (anti-sense). siCont (universal control; STPharm, South Korea) was 5′-AUGAACGUGAAUUGCUCAATT-3′ (sense) and 5′-UUGAGCAAUUCACGUUCAUTT-3′ (anti-sense). siRNAs were transfected at a final concentration of 50 nmole/L for 72 hours with Lipofectamine RNAmaxi (Thermo Fisher Scientific, 13778150) according to the manufacturer’s protocols.

### Western blotting

Western blot was performed as follows. Briefly, whole cell lysates prepared in ice-cold Pierce IP Lysis/Wash Buffer (Thermo Fisher Scientific, 87787) containing protease inhibitor cocktail I (Sigma-Aldrich, P8340), and phosphatase inhibitor cocktail III (Sigma-Aldrich, P0044) were separated by sodium dodecyl sulfate-polyacrylamide gel electrophoresis (SDS-PAGE), transferred onto nitrocellulose membranes (Immobilon-P; Millipore IPVH 08130), blocked with 5% skim milk (BD Biosciences, 232100) in 0.01 M TBS (pH 7.5; Welgene, ML023-03) containing 0.5% Tween 20 (TBST; Biopure, 56-40-6) and blotted with the indicated antibodies. Then, the membranes were reacted with horseradish peroxidase-conjugated goat anti-rabbit-IgG (1:5000; AbFrontier, LF-SA5002a) for 2 hours at room temperature and visualized by WESTSAVE up (AbFrontier, LF-QC0101).

Primary antibodies used for Western blotting were as follows: TIPRL (1:5000, Bethyl, A300-663A), CD133 (1:1000; CST, 5860), LC3B (1:1000; Sigma-Aldrich, L7542), HA-Tag (1:1000, CST, 3724 s) and GAPDH (1:10,000; AbFrontier, LF-PA0212).

### Cell proliferation and survival assays (MTT assay)

To determine effect of TIPRL knockdown on proliferation and survival of Huh7 cells, MTT (M2128, Merck) assay was performed; briefly, 48 hours after siRNAs transfection, the transfected cells were reseeded onto either attached plates (353072, Corning) or suspension plates (7007, Corning), and then the media was replaced with 2 mg/ml MTT solution. Next, the plates were incubated in the dark for additional four hours. MTT solution was removed followed by addition of DMSO (1380, Duksan, South Korea) to dissolve the dark blue formazan precipitates. Optical density was measured using microplate reader (Multiscan Go, Thermo Scientific) at 570 nm.

### Quantitative RT-PCR

RNA extraction and the following reverse-transcription (RT) with 1.0 μg RNA and Oligo (dT)_12-18_ primers were performed using a PureHelix^TM^ Total RNA purification kit (Nanohelix, South Korea) and HelixCript^TM^ First cDNA Synthesis Kit (Nanohelix, South Korea), respectively. A polymerase-chain reaction (PCR) was conducted with the specific primers for the target genes as follows (Supplementary Table 3): the RT and the qPCR were carried out using the GeneAmp PCR system 9700 (Applied Biosystems) and the CFX96TM Real-Time System (Bio-Rad) in a 10 μl reaction mixture containing 1 μl of diluted DNA template, 2 pmole of each primer and either 5 μl of HelixAmp^TM^ Ready-2 × -MultiPlex or RealHelixTM Premier qPCR Kit (Nanohelix, South Korea), respectively. For the control, glyceraldehyde 3-phosphate dehydrogenase was used. qPCR amplification was carried out three times independently. Each PCR product for the target genes was confirmed as a single band of the expected size on 1.5% agarose gel.

### Immunocytochemistry

Cells were seeded at a density of 100,000 cells per ml in an open μ-Slide (ibid, 80826), and then transfected with siRNAs. After 72 hours, cells were fixed/permeabilized using an Image-IT FIX-perm kit (Thermo Fisher Scientific, R37602) and incubated with an antibody against ALDH (1:100; BD, 611194). The primary antibody was detected with Alexa Fluor 488 goat anti-mouse (1:100; Thermo Fisher Scientific, T7458). DAPI was used for staining the nuclei. After that, confocal observation and quantification of expression were performed using the ZEN LSM 800 and the Zen 2.3 lite program (Carl Zeiss), respectively. Briefly, once confocal observation was performed: at least five frames for each sample were obtained. To quantify immunofluorescence of target proteins, we used a ZEN 2.3 lite program (Carl Zeiss) as follows: regions of interest (ROI) were defined using the Histo tool, thereby each group contained an equal numbers of cells. To do that, the integrated density value (IDV) was assessed for the blue channel (DAPI staining). Then, the IDVs were determined for all target proteins provided in the Histo tool. After that, each IDVs were divided by the mean IDV value. Each value was given a log 2 for global normalization.

### Statistical analysis

A multivariate Cox proportional hazard model by XLSTAT version 2017.11.16., was used to determine the impact of TIPRL, LC3, CD133 and CD46 with sex as a covariate on HCCs patients’ survival time and to evaluate the violation of the proportional risk assumption. The public database (www.kmplot.com) was used to compare patient subgroups stratified by mRNA expression for survival outcomes. GraphPad Prism 7 (GraphPad Software) was used to plot the ROC curves and to calculate the corresponding AUC and cut-off as well as sensitivity/specificity.

For *in vitro* study, data was analyzed using GraphPad Prism version 7. The parameters indicating statistical significance with the *p* values are noted within each figure. Error bars in the graphical data represent mean ± standard error of the mean (S.E.M). The data in the figure panels represents independently performed experiments several times on different days. Sample sizes were determined, given previous experience with the assays. Although it was not examined whether the data converges with the assumption of the statistical approach, the assays used in this study were usually mentioned in the literature we used in this study.

### Ethics approval and consent

We gained review exemption from Korea Institutional Review Board; that is, no formal ethics approval was required in this particular case. The study was performed in accordance with the Declaration of Helsinki.

### Consent for publication

All authors agree to publish the paper in its present form.

## Supplementary information

Supplementary information

Supplementary Table 1

Supplementary Table 2

Supplementary Table 3

Supplementary Table 4

Supplementary Table 5

## Data Availability

All the data analyzed or generated in this study are included in this article and its supplementary information file.
