# Supplementary material for: The positive correlation of TIPRL with LC3 and CD133 contributes to cancer aggressiveness: potential biomarkers for early liver cancer
Source: Sci Rep. 2019 Nov 14;9:16802. doi: 10.1038/s41598-019-53191-5 (PMC6856114; doi:10.1038/s41598-019-53191-5)
Supplement: Supplementary file 1 — Supplementary information [file 41598_2019_53191_MOESM1_ESM.pdf]

Supplemental Information

The positive correlation of TIPRL with LC3 and CD133 contributes to cancer aggressiveness: potential biomarkers for early liver cancer

Soo Young Jun[1,2,5**], Su-Jin Jeon[1,2,5**], Ji-Yong Yoon[1,2], Jeong-Ju Lee[1,2], Hyang Ran Yoon[3], Min-Hyuk Choi[1,2,5], Debasish Halder[1,2], Kwangho Lee[4,5] and Nam-Soon Kim[1,2,5*]

[1]Medical Genomics Research Center, [2]Rare-Disease Research Center, Division of Strategic Research Groups, [3]Immunotherapy Convergence Research Center, Korea Research Institute of Bioscience & Biotechnology, Daejeon 34141, Korea

[4] Bio and Drug Discovery Division, Korea Research Institute of Chemical Technology, Daejeon, 34114, Republic of Korea.

[5]Functional Genomics, University of Science & Technology, Daejeon 34113, Korea

Supplementary Figure 1. Upregulated levels of TIPRL, LC3 and CD133 in HCC tissue. (a-c) Human HCC tissues (Supplementary Tables 1-2) were stained with the indicated antibodies followed by confocal observation. Each set comprised of normal and HCC tissues. **(d)** As a positive control, lung tissue, provided from US Biomax, was simultaneously stained and presented. DAPI was used for nucleus staining, and scar bar, 100 μm. The Oncomine database (www.oncomine.org) was used to determine the mRNA levels of *TIPRL* **(e)** and of *MAP1LC3A* **(f)** in two different cohorts, Wurmbach and TCGA. Statistical significance (**, $P<0.01$; ***, $P<0.0001$) was determined by an unpaired t test with Welch's correction, and the % differences are shown. (n), the number of samples.

Supplementary Figure 2. TIPRL modulates LC3 and CD133 expression, thereby contributing to tumor aggressiveness. Huh7 **(a-b, e, g, i)** and SK-Hep-1 **(c-d, f, h, j)** were cultured in ultra-low affinity plates. Quantitative RT-PCR analyses were performed **(a-d)** using cells transfected with second siTIPRL and specific primers (Supplementary Table 3). Ectopic *TIPRL* was dose-dependently overexpressed in siTIPRL- and siCD133-transfected Huh7 **(e, g, i)** and in siTIPRL-SK-Hep-1 **(f, h, j)** cells. **(e, f)** qRT-PCR, **(g, h)** Western blot and **(I, j)** MTT analyses were performed. For a loading control, GAPDH was used. Statistical significance (**, $P<0.01$; ***, $P<0.0001$) was calculated by 2way ANOVA. n=4.

Supplementary Figure 3. Diagnostic assessments of TIPRL, LC3, CD133 and the TIPRL/LC3/CD133 models in liver cancers. (a-b) Diagnostic efficacies of TIPRL, LC3, CD133 and the TIPRL/LC3/CD133 models in liver cancers were calculated using ROC analysis. AUC, area under the curve.

Supplementary Figure 4. A multivariate Cox hazard regression analysis for the TIPRL/LC3/CD46/CD133/sex model in HCCs. (a) Hazard effect and independency of the variables, TIPRL, LC3, CD133, CD46, sex (male vs female), on survivability of HCC patients using XLSTAT were determined by a Cox regression hazard model (upper) and a proportionality test (lower).

Supplementary Table 1. Clinicopathological features of training set liver cancer tissues. Human tissues were categorized by patients' information provided by US Biomax. The average age of patients and numbers of each category are shown. ± calculated; % presented.

Supplementary Table 2. Expression levels of TIPRL, LC3 and CD133 as well as clinicopathological information. Human tissues and patients' information were provided from US Biomax. Tissues were stained with the indicated antibodies, and then confocal observation was performed. Each expression was quantified using a ZEN 2.3 lite (Carl Zeiss) program and global normalization.

Supplementary Table 3. Primers used for quantitative RT-PCR. Primers for conducting quantitative RT-PCR were designed using Primer 3 program. Each PCR product for the target genes was confirmed as a single band of the expected size on 1.5% agarose gel.

Supplementary Table 4. Clinopathological features of validating set HCC tissues. A different set of human tissues were categorized by patients' information provided by US Biomax. The average age of patients and numbers of each category are shown. ± calculated; % presented.

Supplementary Table 5. Levels of TIPRL, LC3, CD133 and CD46 as well as patients' clinicopathological information. Human tissues and patients' information used in Supplementary Table 4 were provided from US Biomax. Tissues were stained with the indicated antibodies followed by confocal observation. Each expression was quantified using a ZEN 2.3 lite (Carl Zeiss) program and global normalization.

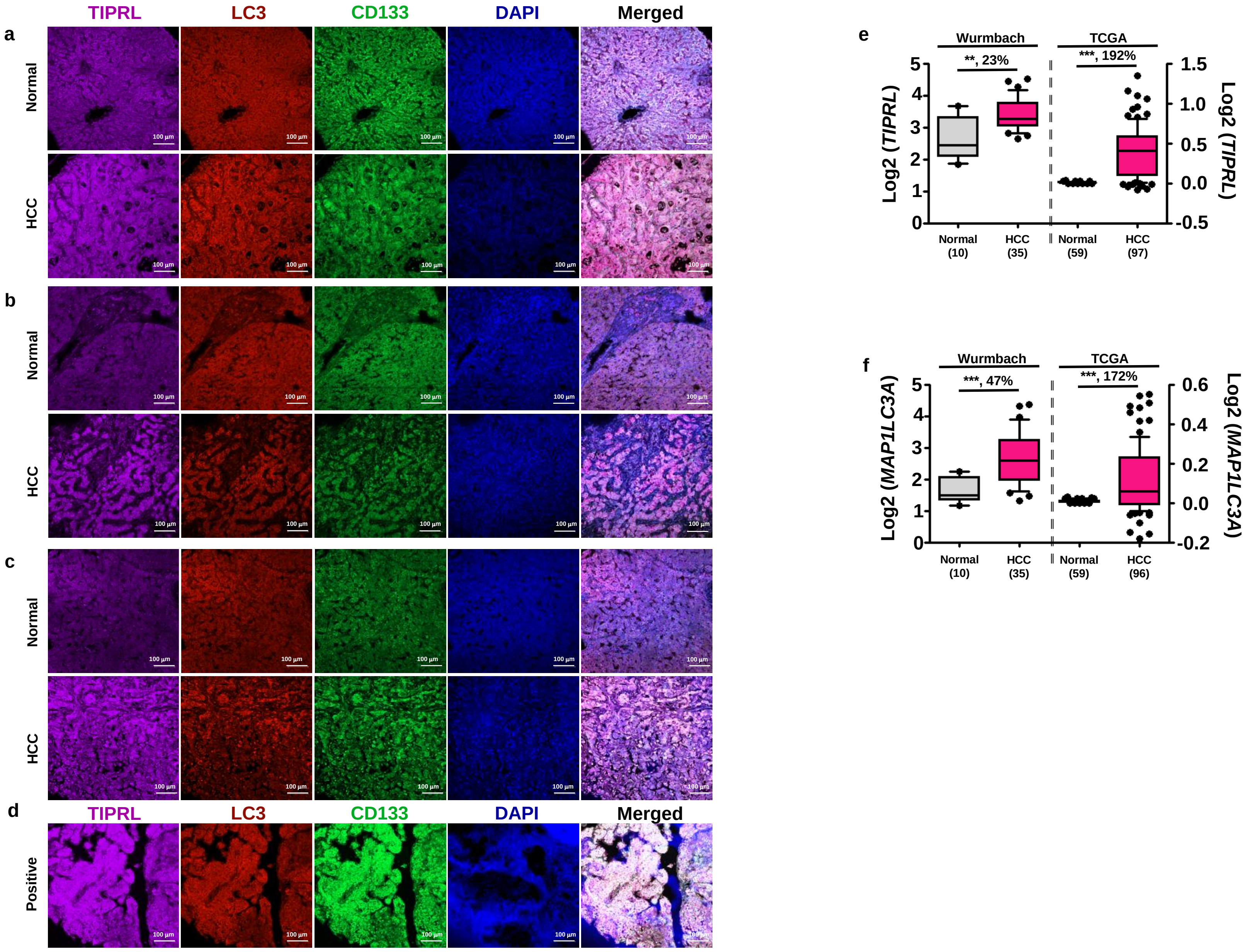

Supplementary Figure 1. Upregulated levels of *TIPRL, LC3* and CD133 in liver cancers

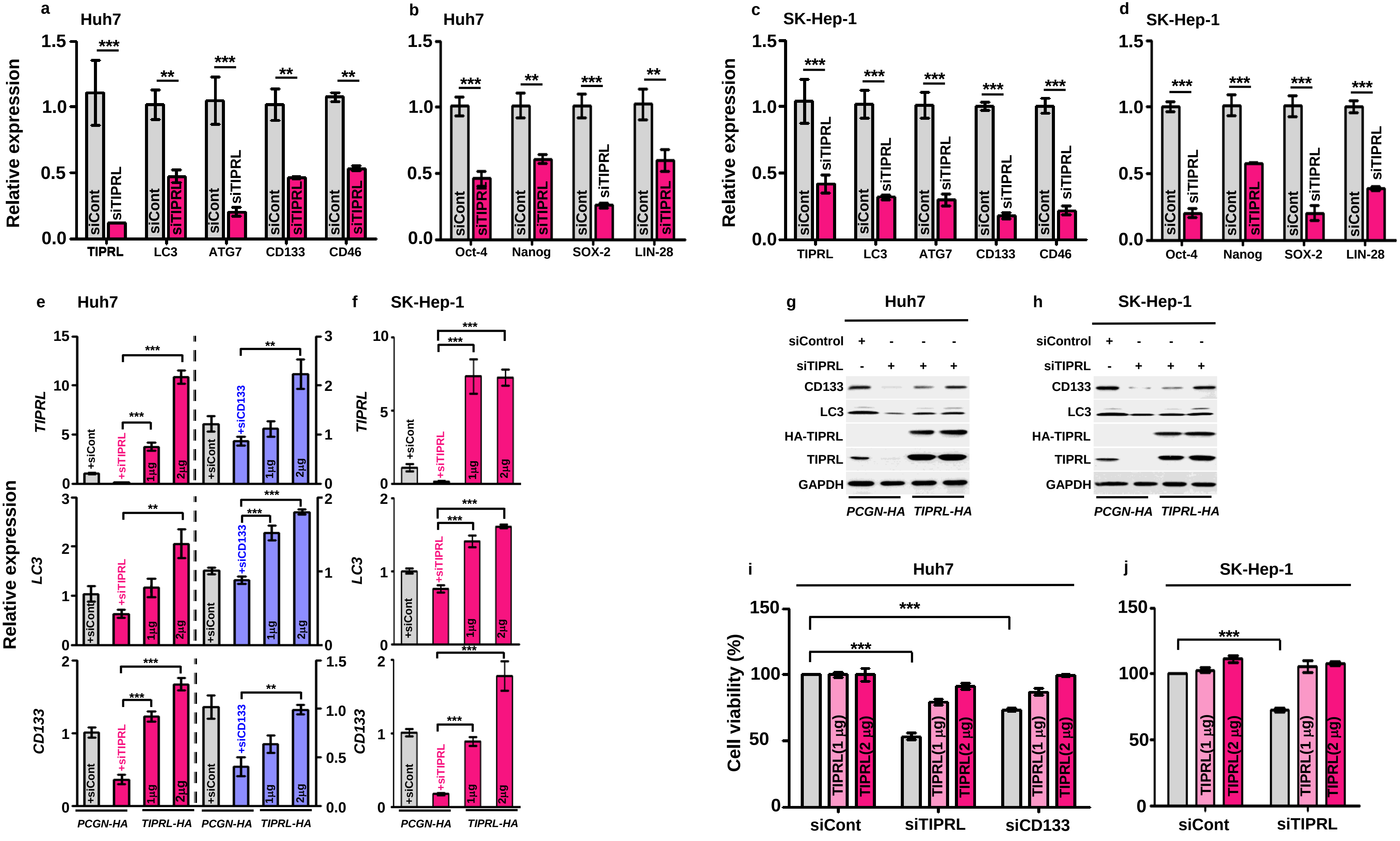

Supplementary Figure 2. TIPRL modulates LC3 and CD133 expression, thereby contributing to tumor aggressiveness.

a

Liver cancers	Incidence					
	AUC (95% CI, %)			cut-off (95% CI, %)		
TIPRL	52%	Sensitivity	54.2 (48.5-59.9)	-1	Sensitivity	9.7 (6.7-13.6)
		Specificity	50.0 (27.2-72.8)		Specificity	95.0 (75.1-99.9)
LC3	54%	Sensitivity	59.1 (53.4-64.6)	-0.82	Sensitivity	18.2 (14.0-23.0)
		Specificity	55.0 (31.5-76.9)		Specificity	95.0 (75.1-99.9)
CD133	51%	Sensitivity	51.6 (45.9-57.3)	0.26	Sensitivity	30.5 (25.4-36.0)
		Specificity	50.0 (27.2-72.8)		Specificity	85.0 (62.1-96.8)
TIPRL/LC3/CD133	53%	Sensitivity	56.8 (51.1-62.4)	-0.82	Sensitivity	17.5 (13.5-22.3)
		Specificity	55.0 (31.5-76.9)		Specificity	95.0 (75.1-99.9)

b

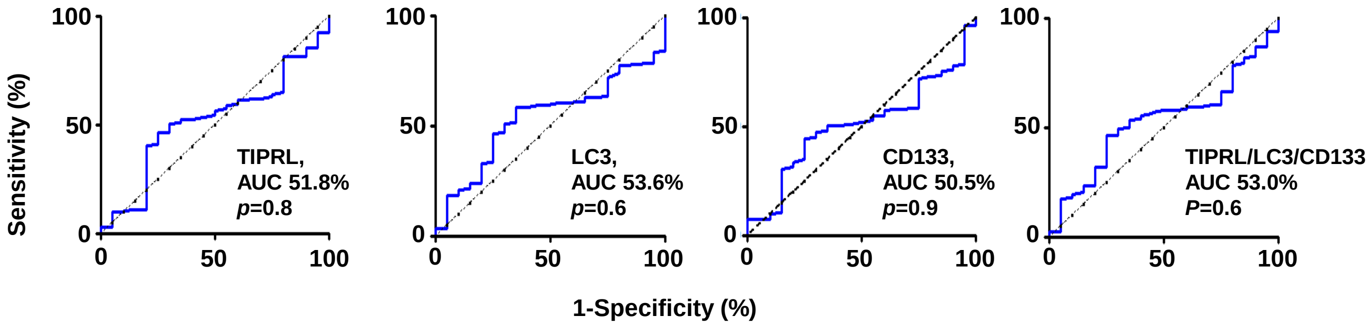

Supplementary Figure 3. Diagnostic assessments of TIPRL, LC3, CD133 and the TIPRL/LC3/CD133 models in liver cancers

a

Regression coefficients:

Predictor Variable	Hazard Ratio (95%CI)	*p value*
TIPRL	14.224 (0.9-222.5)	0.06
LC3	0.021 (0.0-0.5)	0.02
CD46	0.876 (0.5-1.5)	0.6
CD133	2.152 (0.9-5.0)	0.08
sex (male vs female)	5.703 (2.8-11.6)	<0.0001

Proportionality test:

Variable	rho	Chi-square	*p value*
TIPRL	0.0061795	0.0061495	0.937495
LC3	0.0111922	0.0192947	0.889525
CD46	0.0176501	0.0378357	0.8457733
CD133	-0.18377	2.4245886	0.1194446
sex (male vs female)	0.3425474	7.2772944	0.0069832
TIPRL/LC3/CD46/CD133/sex		12.929882	0.0240448

Supplementary Figure 4. A multivariate Cox hazard regression analysis for the TIPRL/LC3/CD46/CD133/sex model in HCCs
